# 气相色谱-三重四极杆质谱动态多反应监测模式测定枸杞干果中118种农药残留

**DOI:** 10.3724/SP.J.1123.2020.07028

**Published:** 2021-06-08

**Authors:** Zhimin YANG, Wen ZHANG, Fuxiang WU, Xingzhi WANG, Xiaohui XU

**Affiliations:** 兰州市食品药品检验检测研究院, 甘肃 兰州 730050; Lanzhou Institutes for Food and Drug Control, Lanzhou 730050, China; 兰州市食品药品检验检测研究院, 甘肃 兰州 730050; Lanzhou Institutes for Food and Drug Control, Lanzhou 730050, China; 兰州市食品药品检验检测研究院, 甘肃 兰州 730050; Lanzhou Institutes for Food and Drug Control, Lanzhou 730050, China; 兰州市食品药品检验检测研究院, 甘肃 兰州 730050; Lanzhou Institutes for Food and Drug Control, Lanzhou 730050, China; 兰州市食品药品检验检测研究院, 甘肃 兰州 730050; Lanzhou Institutes for Food and Drug Control, Lanzhou 730050, China

**Keywords:** 气相色谱-三重四极杆质谱, 动态多反应监测, 农药残留, 枸杞干果, gas chromatography-triple quadrupole mass spectrometry (GC-MS/MS), dynamic multiple reaction monitoring (dMRM), pesticide residue, dried wolfberry

## Abstract

枸杞中丰富的营养物质深受广大消费者喜爱,但也极易受到病虫侵害,农药残留问题引起了人们的广泛关注。基质干扰是微量分析的一个难点,高灵敏度和高选择性的色谱-串联质谱技术是复杂基质中微量分析强有力的工具,动态扫描监测模式的优越性逐渐取代传统的多反应监测扫描模式,简便、快速、省时的QuEChERS前处理方法已被广泛应用于食品的农药残留检测中。采用改良QuEChERS法结合动态多反应监测模式(dMRM),建立了同时检测枸杞干果中118种农药残留的气相色谱-三重四极杆质谱分析方法。实验比较了不同加水量、提取溶剂、提取过程中温度提取条件,以及无水硫酸镁吸水剂、乙二胺-*N*-丙基硅烷化硅胶PSA、十八烷基硅烷键合硅胶C_18_净化填料添加量时农药的回收率,确定出最优前处理方法。结果表明,5 g样品经10 mL超纯水复水,用10 mL乙腈浸提,于-18 ℃冷冻10 min后,用缓冲体系盐包提取后,经800 mg无水硫酸镁、150 mg PSA、150 mg C_18_混合填料净化,基质匹配外标法定量。118种农药在一定范围内线性关系良好,相关系数*R*^2^≥0.9923,检出限和定量限分别为0.006~28.344 μg/kg和0.021~94.480 μg/kg, 4个添加水平的回收率为64.97%~126.21%, RSDs均小于19%(*n*=6)。基质效应考察结果表明,82%的农药呈现为基质增强效应,其他为基质抑制效应;9%的农药表现为强基质效应,其他为中等或弱基质效应;采用基质匹配标准曲线校正,可有效降低基质效应的影响。应用建立的方法测定10批枸杞样品,全部样品有农药检出,共检出农药22种。该方法操作简便快速,准确可靠,适用于枸杞干果中农药多残留的日常检测和快速筛查。

## Abstract (en)

Wolfberry fruit is very popular among consumers because it is rich in nutrients. However, it is vulnerable to diseases caused by insect pest feeding and microbial pathogen infection. Pesticide application is the main approach for controlling wolfberry disease; however, various concerns have been raised regarding chemical residues in foodstuffs and consequent environmental contamination. Matrix interference is a significant challenge in trace analysis. Chromatography, coupled with MS techniques with high sensitivity and selectivity, proved to be a powerful tool for the detection of multi-pesticide residues in complex matrices. The traditional MRM mode has been gradually replaced by the dynamic MRM (dMRM) mode, which could dynamically allocate the retention time window of each target pesticide, significantly adjust the loading cycle time of multiple compounds, and improve the analysis efficiency. The QuEChERS pretreatment method, based on dispersive solid-phase extraction, has been widely used in the detection of pesticide residues in food because it is simple and rapid. In this study, a robust and high-throughput method was established for the simultaneous determination of 118 pesticide residues in wolfberry using the modified QuEChERS method, combined with gas chromatography-triple quadrupole mass spectrometry in dMRM mode. The optimal pretreatment method was determined by comparing the recovery rates obtained with different volumes of added water (5, 10, 15, and 20 mL), different extraction solvents (acetone, *n*-hexane, acetonitrile, and acetonitrile containing 0.1% formic acid), different extraction temperatures (normal temperature, -18 ℃ for 10 min and 20 min), water absorbent (anhydrous magnesium sulfate), and purification with primary secondary amine (PSA) and octadecylsilane (C_18_). The results showed that 5 g samples were rehydrated with 10 mL ultrapure water, extracted with 10 mL acetonitrile, frozen at -18 ℃ for 10 min, partitioned with buffer system salt package containing 4.0 g anhydrous magnesium sulfate, 1.0 g sodium chloride, 1.0 g sodium citrate, and 0.5 g disodium citrate, purified up with 800 mg MgSO_4_, 150 mg PSA, and 150 mg C_18_. Pesticides were separated on a capillary column HP-5MS UI (30 m×0.25 mm×0.25 μm), and quantified by a matrix-matched external standard method. The results showed that the 118 pesticides exhibited good linearity in the range from 20 to 640 μg/L, with correlation coefficients *R*^2^≥0.9923. The limits of detection and quantification were 0.006-28.344 μg/kg and 0.021-94.480 μg/kg, respectively. The average recoveries at four spiked levels of 0.01, 0.04, 0.10, and 0.20 mg/kg were in the range of 64.97%-126.21%, with relative standard deviations (RSDs) of 0.69%-18.86% (*n*=6). The results of the matrix effect showed that 82% of the pesticides exhibited matrix enhancement effects, while others showed matrix inhibition effects. In addition, 9% of the pesticides showed a strong matrix effect, while others showed moderate or weak matrix effects. The matrix effects could be reduced by the matrix-matched standard curve method. The proposed method was employed for the analysis of 10 real samples purchased from local markets. The results demonstrated that pesticides were detected in all the samples, 22 pesticides were detected in total, and 3-12 pesticides were found in a single sample. Chlorpyrifos, fipronil, cypermethrin, pyridaben, and difenoconazole were detected at high detection rates. The captan content in a batch of samples was 1.4066 mg/kg. Thus, the optimized method is simple, fast, accurate, and reliable, and it is suitable for the routine detection and rapid screening of the multi-pesticide residues in wolfberry.

枸杞是茄科多年生落叶灌木,其干燥成熟果实枸杞子是国家卫健委公布的药食同源物质之一^[1]^。枸杞中含有多糖、类胡萝卜素、维生素、脂肪酸、酚类、黄酮等成分^[2,3,4]^,丰富的营养深受广大消费者喜爱,但也极易受到病虫侵害。生产者为了确保产量,必须使用化学农药进行防治,但是长期用药会使一些害虫和病原微生物产生抗药性,农户常常采取加大农药浓度或者增加喷洒次数等方式对病虫害进行防治。这些不规范的用药方式是造成农药残留的主要因素。农药残留问题不仅影响枸杞产品的质量,给人体健康带来安全隐患,目前也成为我国枸杞的出口贸易壁垒^[5,6]^。农药多残留快速检测方法的开发是市场监管开展日常监督检验和风险评估的主要筛查技术。因此,建立枸杞基质中农药多残留快速灵敏的检测方法,对保护消费者的安全、推动枸杞产业的可持续发展具有重要意义。

目前,枸杞中农药残留检测技术主要有气相色谱法^[7,8]^、气相色谱-串联质谱法^[9,10,11]^、液相色谱法^[12]^、液相色谱-串联质谱法^[13,14]^等。气相色谱和液相色谱检测技术定量准确,但在农药多残留测定时易受基质干扰,在灵敏度和确证方面存在不足。色谱与质谱联用法可降低基质干扰,在灵敏度和选择性方面具有优势,已成为农药多残留分析的主要方法。鉴于气相色谱-三重四极杆质谱法高准确性、高效性及基质干扰小的特点,以及动态多反应监测(dMRM)模式检测的高灵敏度、高选择性和对色谱峰形改善等优点,气相色谱-三重四极杆质谱结合dMRM,成为高通量筛查农药多残留的首选方法。关于枸杞中农药残留检测方法的报道,目前涉及的农药数量最多为几十种,虽然GB 23200.10-2016中检测农药上百种,但是以传统的固相萃取法进行前处理,过程复杂耗时,不适用于快速筛查。QuEChERS前处理方法具有经济环保、简便快速、准确高效等特点,近年来已被广泛用于复杂基质中农药多残留的测定。

运用气相色谱-质谱仪的动态多反应扫描模式监测枸杞中的农药多残留检测方法鲜见报道。由于基质的差异性,本文参考QuEChERS法测定果蔬中农药多残留的国标法^[15]^,从提取、净化等前处理条件结合气相色谱-三重四极杆质谱仪所备有的检测功能,优化出可同时快速测定枸杞中农药多残留的检测方法,为保证枸杞质量安全提供技术支撑。

## 1 实验部分

### 1.1 仪器、试剂与材料

7890B-7000D气相色谱-三重四极杆质谱仪(美国Agilent公司);5810R高速低温离心机(德国Eppendorf公司);VORTEX-5涡旋混匀器(中国其林贝尔仪器制造有限公司);A11基本型分析研磨机、KS501圆周振荡摇床(德国IKA公司);MS205DU、BSA224S-CW电子天平(德国Sartorius公司);EVA32多功能样品浓缩仪(北京普立泰科仪器有限公司);Milli-Q超纯水机(美国Millipore公司)。

118种农药标准品(纯度均≥95.0%,德国Dr. Ehrenstorfer公司、北京郑翔科技有限公司);甲酸、正己烷、乙腈(色谱纯,德国Merck公司);丙酮(色谱纯,科密欧化学试剂有限公司);无水硫酸镁、氯化钠、柠檬酸钠和柠檬酸二钠(分析纯,国药集团化学试剂有限公司);乙二胺-*N*-丙基硅烷化硅胶(PSA)、十八烷基硅烷键合硅胶(C_18_)(美国Agilent公司)。

### 1.2 分析条件

色谱柱:HP-5MS UI气相色谱柱(30 m×0.25 mm×0.25 μm);进样口温度:280 ℃;传输线温度:280 ℃;载气:氦气;流速:0.9 mL/min;程序升温:初始温度60 ℃,保持1 min,以40 ℃/min的速率升温至170 ℃,再以10 ℃/min的速率升温至310 ℃,保持3 min;进样量:1.0 μL,不分流进样。

离子源:EI源;离子源温度:230 ℃;动态多反应监测扫描模式;电子能量:70 eV;溶剂延迟:2.5 min;采集软件:Agilent MassHunter。118种农药的质谱条件具体见表1。

**表 1:** 118种农药的保留时间与质谱参数

No.	Compound	Retention time/min	Ion pairs/(m/z)	Collision energies/eV
1	dichlorvos (敌敌畏)	4.64	184.9>93.0, 109.0>79.0	15, 5
2	carbofuran (克百威)	4.91	164.2>149.1, 149.1>121.1	10, 5
3	captan (克菌丹)	5.91	151.0>80.0, 151.0>79.0	5, 15
4	carbaryl (甲萘威)	6.14	144.0>116.1, 144.0>115.1	10, 20
5	molinate (禾草敌)	6.28	126.2>98.1, 126.2>55.1	5, 10
6	tecnazene (四氯硝基苯)	6.78	258.9>201.0, 214.9>179.0	10, 10
7	hexaflumuron (氟铃脲)	6.80	277.0>176.0, 277.0>148.0	15, 30
8	diphenylamine (二苯胺)	6.85	169.0>168.2, 168.0>167.2	15, 15
9	ethoprophos (灭线磷)	6.89	157.9>114.0, 157.9>97.0	5, 15
10	chlorpropham (氯苯胺灵)	6.99	171.0>127.1, 127.0>65.1	5, 25
11	trifluralin (氟乐灵)	7.11	305.9>264.0, 264.0>160.1	5, 15
12	sulfotep (治螟磷)	7.24	321.8>145.8, 201.8>145.9	25, 10
13	cadusafos (硫线磷)	7.29	158.8>97.0, 126.9>98.9	15, 5
14	phorate (甲拌磷)	7.36	260.0>75.0, 121.0>47.0	5, 30
15	α-hexachlorocyclohexane (α-六六六)	7.47	218.9>183.0, 216.9>181.0	5, 5
16	hexachlorobenzene (六氯苯)	7.63	283.8>248.8, 283.8>213.9	15, 30
17	dicloran (氯硝胺)	7.67	160.1>124.1, 124.1>73.0	10, 10
18	γ-hexachlorocyclohexane (γ-六六六)	7.79	216.9>181.0, 181.0>145.0	5, 15
19	β-hexachlorocyclohexane (β-六六六)	7.90	216.9>181.0, 181.0>145.0	5, 15
20	terbufos (特丁硫磷)	7.99	230.9>175.0, 230.9>129.0	10, 20
21	propyzamide (炔苯酰草胺)	8.02	175.0>147.0, 173.0>145.0	15, 15
22	trichlorfon (敌百虫)	8.06	109.0>81.0, 109.0>63.0	6, 10
23	quintozene (五氯硝基苯)	8.06	295.0>236.8, 248.8>213.8	20, 15
24	fonofos (地虫硫磷)	8.08	245.9>109.0, 136.9>109.0	15, 5
25	pyrimethanil (嘧霉胺)	8.12	198.0>183.1, 198.0>118.0	15, 35
26	diazinon (二嗪磷)	8.12	137.1>84.0, 137.1>54.0	10, 20
27	δ-hexachlorocyclohexane (δ-六六六)	8.35	217.0>181.1, 181.1>145.1	5, 15
28	pirimicarb (抗蚜威)	8.53	238.0>166.2, 166.0>55.1	10, 20
29	phosphamidon (磷胺)	8.76	226.9>127.0, 127.0>109.0	5, 10
30	vinclozolin (乙烯菌核利)	8.92	197.9>145.0, 187.0>124.0	15, 20
31	chlorpyrifos-methyl (甲基毒死蜱)	8.95	287.9>92.9, 285.9>92.9	20, 20
32	parathion-methyl (甲基对硫磷)	8.95	262.9>109.0, 232.9>109.0	30, 10
33	tolclofos-methyl (甲基立枯磷)	9.03	265.0>250.0, 265.0>93.0	15, 25
34	metalaxyl (甲霜灵)	9.12	192.0>160.1, 160.0>145.1	5, 10
35	heptachlor (七氯)	9.13	273.7>238.9, 271.7>236.9	25, 25
36	paraoxon (对氧磷)	9.13	148.9>119.0, 108.9>91.0	5, 5
37	isazofos (氯唑磷)	9.35	161.0>146.0, 161.0>119.1	5, 5
38	fenitrothion (杀螟硫磷)	9.39	277.0>260.1, 277.0>109.0	5, 20
39	malathion (马拉硫磷)	9.53	172.9>99.0, 126.9>99.0	15, 5
40	fenthion (倍硫磷)	9.71	278.0>109.0, 124.9>79.0	15, 5
41	aldrin (艾氏剂)	9.72	262.9>192.9, 254.9>220.0	35, 20
42	chlorpyrifos (毒死蜱)	9.75	314.0>286.0, 314.0>258.0	20, 15
43	parathion (对硫磷)	9.77	290.9>109.0, 138.9>109.0	10, 5
44	triadimefon (三唑酮)	9.78	208.0>181.1, 208.0>111.0	5, 20
45	dicofol (三氯杀螨醇)	9.83	250.9>138.9, 139.0>111.0	15, 15
46	isocarbofos (水胺硫磷)	9.87	135.9>108.0, 120.0>92.0	15, 10
47	cyprodinil (嘧菌环胺)	10.18	225.2>224.3, 224.2>208.2	10, 20
48	isofenphos-methyl (甲基异柳磷)	10.19	241.0>121.0, 199.0>121.0	15, 15
49	pendimethalin (二甲戊灵)	10.28	251.8>162.2, 251.8>161.1	10, 15
50	penconazole (戊菌唑)	10.31	248.0>192.1, 248.0>157.1	15, 25
51	tolylfluanid (甲苯氟磺胺)	10.40	237.9>137.0, 136.9>91.1	25, 25
52	fipronil (氟虫腈)	10.45	350.7>254.9, 254.8>228	15, 15
53	triadimenol (三唑醇)	10.50	168.0>70.0, 128.0>65.0	10, 25
54	zoxamide (苯酰菌胺)	10.57	189.0>161.1, 187.0>159.1	15, 15
55	procymidone (腐霉利)	10.61	282.8>96.0, 282.8>68.1	10, 15
56	triflumizole (氟菌唑)	10.62	206.0>186.0, 206.0>179.0	10, 15
57	haloxyfop-methyl (氟吡甲禾灵)	10.72	375.0>316.0, 316.0>91.0	10, 20
58	methidathion (杀扑磷)	10.77	144.9>85.0, 144.9>58.1	5, 15
59	chlordane (氯丹)	10.78	372.9>265.9, 271.9>236.9	20, 15
60	fenothiocarb (精噁唑禾草灵)	10.84	160.1>72.1, 72.0>56.0	10, 10
61	o,p'-dichlorodiphenyldichloroethylene (o,p'-滴滴伊)	10.84	248.0>176.2, 246.0>176.2	30, 30
62	flumetralin (氟节胺)	10.96	143.0>117.0, 143.0>107.1	20, 20
63	picoxystrobin (啶氧菌酯)	11.07	145.0>115.1, 145.0>102.1	15, 25
64	fenamiphos (苯线磷)	11.10	217.0>202.1, 154.0>139.0	10, 10
65	hexaconazole (己唑醇)	11.19	256.0>159.0, 231.0>175.0	15, 10
66	profenofos (丙溴磷)	11.31	338.8>268.7, 207.9>63.0	15, 30
67	pretilachlor (丙草胺)	11.33	162.1>147.2, 162.1>132.2	10, 20
68	p,p'-dichlorodiphenyldichloroethylene (p,p'-滴滴伊)	11.39	315.8>246.0, 246.1>176.2	15, 30
69	dieldrin (狄氏剂)	11.47	277.0>241.0, 262.9>193.0	5, 35
70	myclobutanil (腈菌唑)	11.48	179.0>125.1, 150.0>123.0	10, 15
71	flusilazole (氟硅唑)	11.52	314.7>232.9, 233.0>165.1	10, 15
72	o,p'-dichlorodiphenyldichloroethane (o,p'-滴滴滴)	11.54	237.0>165.2, 235.0>165.2	20, 20
73	fipronil-sulfone (氯氟氰菊酯)	11.54	384.8>256.8, 382.8>254.9	20, 20
74	thifluzamide (噻呋酰胺)	11.58	193.9>166.0, 193.9>124.9	10, 25
75	chlorfenapyr (虫螨腈)	11.80	246.9>227.0, 136.9>102.0	15, 15
76	endrin (异狄氏剂)	11.86	262.8>193.0, 244.8>173.0	35, 30
77	p,p'-dichlorodiphenyldichloroethane (p,p'-滴滴滴)	12.13	236.9>165.2, 234.9>165.1	20, 20
78	o,p'-dichlorodiphenyltrichloroethane (o,p'-滴滴涕)	12.20	237.0>165.2, 235.0>165.2	20, 20
79	clethodim (烯草酮)	12.34	205.0>176.0, 164.0>81.0	15, 25
80	triazophos (三唑磷)	12.41	161.2>134.2, 161.2>106.1	5, 10
81	benalaxyl (苯霜灵)	12.59	148.0>105.1, 148.0>77.0	20, 35
82	edifenphos (敌瘟磷)	12.67	201.0>109.0, 172.9>109.0	10, 5
83	trifloxystrobin (肟菌酯)	12.71	172.0>145.1, 116.0>89.0	15, 15
84	p,p'-dichlorodiphenyltrichloroethane (p,p'-滴滴涕)	12.78	235.0>199.1, 235.0>165.1	15, 20
85	propiconazole (丙环唑)	12.78	172.9>145.0, 172.9>74.0	15, 45
86	tebuconazole (戊唑醇)	13.00	250.0>125.0, 125.0>89.0	20, 15
87	propargite (炔螨特)	13.05	135.0>107.1, 135.0>77.1	10, 30
88	piperonyl butoxide (增效醚)	13.12	176.1>131.1, 176.1>103.1	15, 25
89	bioresmethrin (生物苄呋菊酯)	13.15	143.0>128.1, 123.0>81.1	10, 8
90	azinphos-methyl (谷硫磷)	13.63	160.0>77.0, 160.0>50.9	16, 34
91	phosmet (亚胺硫磷)	13.65	160.0>133.1, 160.0>77.1	10, 20
92	bromopropylate (溴螨酯)	13.66	185.0>157.0, 183.0>155.0	15, 15
93	bifenthrin (联苯菊酯)	13.67	181.1>165.1, 166>165.1	20, 25
94	bifenazate (联苯肼酯)	13.70	184.0>169.2, 184.0>141.1	10, 20
95	fenpropathrin (甲氰菊酯)	13.79	264.9>210.0, 208.0>181.1	10, 15
96	fenazaquin (喹螨醚)	13.93	160.0>145.2, 145.0>117.1	5, 10
97	phosalone (伏杀硫磷)	14.33	182.0>111.0, 182.0>75.1	15, 30
98	pyriproxyfen (吡丙醚)	14.37	136.1>96.0, 136.1>78.1	15, 20
99	cyhalothrin (氯氟氰菊酯)	14.64	207.9>181.1, 196.9>141.1	20, 20
100	mirex (灭蚁灵)	14.66	273.8>238.8, 271.8>236.8	15, 15
101	dimethrin (苄菊酯)	15.45	183.1>168.1, 183.1>165.1	10, 10
102	permethrin (氯菊酯)	15.48	162.9>127.1, 162.9>91.1	10, 20
103	pyridaben (哒螨灵)	15.48	147.2>132.2, 147.2>117.1	10, 20
104	coumaphos (蝇毒磷)	15.61	361.9>109.0, 210.0>182.0	15, 10
105	prochloraz (咪鲜胺)	15.65	310.0>69.8, 180.0>138.0	15, 10
106	fenbuconazole (腈苯唑)	15.94	128.9>102.1, 128.9>78.0	15, 20
107	cyfluthrin (氟氯氰菊酯)	16.00	162.9>127.0, 162.9>90.9	5, 15
108	boscalid (啶酰菌胺)	16.31	140.0>112.0, 140.0>76.0	10, 25
109	cypermethrin (氯氰菊酯)	16.33	163.0>127.0, 163.0>91.0	10, 15
110	quizalofop-ethyl (喹禾灵)	16.38	371.8>298.9, 163.0>136.0	10, 10
111	flucythrinate (氟氰戊菊酯)	16.44	199.1>157.0, 199.1>107.1	20, 20
112	etofenprox (醚菊酯)	16.52	163.0>135.1, 163.0>107.1	10, 20
113	tau-fluvalinate (氟胺氰菊酯)	17.32	249.9>200.2, 249.9>55.1	20, 20
114	fenvalerate (氰戊菊酯)	17.33	181.0>151.8, 167.0>125.2	20, 5
115	difenoconazole (苯醚甲环唑)	17.59	322.9>265.0, 264.9>201.9	15, 20
116	deltamethrin (溴氰菊酯)	17.84	252.9>93.0, 252.8>172.0	25, 20
117	azoxystrobin (嘧菌酯)	18.11	344.1>182.9, 344.1>155.8	25, 40
118	dimethomorph (烯酰吗啉)	18.13	302.9>164.9, 300.9>165.0	10, 10

### 1.3 实验方法

1.3.1 标准溶液配制

准确称取各供试标准品,据其溶解性用丙酮或正己烷分别配制成1.0 mg/mL的标准储备液,于4 ℃避光保存备用;混合标准溶液临用前用正己烷稀释。

1.3.2 样品前处理

取枸杞干果样品,于-18 ℃冷冻48 h后立即粉碎,精密称取5 g(精确至0.001 g)样品,置于50 mL离心管中,加入10 mL超纯水振荡混匀,再加入10.00 mL乙腈剧烈振荡10 min,于-18 ℃冷冻10 min,加入4.0 g无水硫酸镁、1.0 g氯化钠、1.0 g柠檬酸钠、0.5 g柠檬酸二钠,立即涡旋混匀1 min,于4 ℃以3900 r/min离心5 min,上清液待净化。

精密移取6.00 mL上清液,移至内含PSA 150 mg、C_18_ 150 mg、无水硫酸镁800 mg的净化管中,涡旋混匀1 min,于4 ℃以3900 r/min离心5 min,精密吸取2.00 mL上清液,置于离心管中,于40 ℃水浴中氮吹至近干,加入1.00 mL正己烷复溶,过0.2 μm有机滤膜,待测定。

1.3.3 基质混合标准溶液的配制

取枸杞空白样品,依照1.3.2节方法制备得到空白基质溶液。临用前用空白基质溶液稀释118种农药混合标准溶液,配制成基质混合标准溶液。

## 2 结果与讨论

### 2.1 dMRM模式下农药残留检测方法的建立

利用美国国家标准和技术研究所(NIST)质谱数据库检索各个目标农药的特征离子碎片和碰撞能量,通过多反应监测模式采集获得每个化合物的保留时间,针对个别化合物采用自动或手动方式进一步优化,据已获得的参数信息建立基于动态多反应监测模式的农药多残留检测方法。该检测方法可以自动对保留时间窗口变化范围进行分配,进而改善多种化合物的负载循环时间,提高分析灵敏度。动态多反应监测模式下118种农药的提取离子流图(见图1)。

**图 1:**
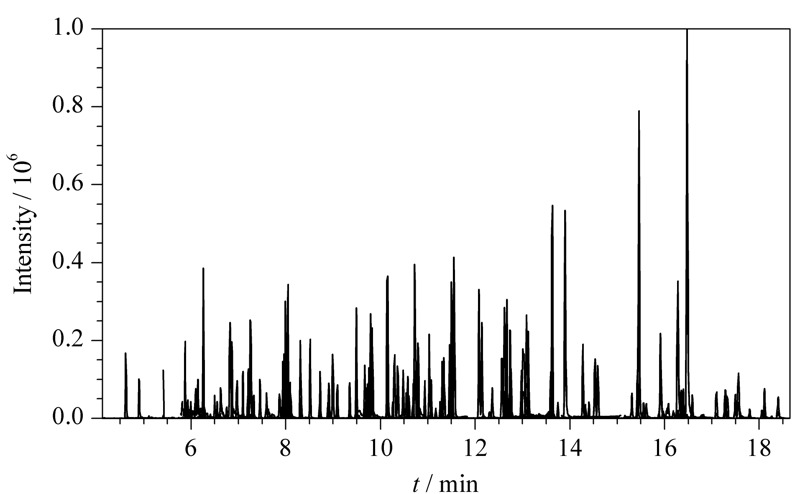
枸杞基质中118种农药的提取离子流图

### 2.2 提取条件的优化

以枸杞干果为基质,在提取过程中分别从样品加水量、提取溶剂以及提取过程中温度的控制考察各个农药的提取效果。

2.2.1 样品加水量的考察

从含水量低的样品中提取农药时,用溶剂直接浸提难以充分渗透到组织内部,加入适量的水进行复水处理有利于提高目标农药的回收率。实验比较了5、10、15、20 mL加水量对目标农药回收率的影响(见图2a)。结果表明,当加水量为5 mL时,回收率小于70%的农药数量较多;当加水量为10 mL时,回收率在70%~110%的数量最多;当加水量超过10 mL时,回收率高于110%的农药数量明显增多,且20 mL加水量的高回收率农药数量较15 mL加水量的多。因此,5 g枸杞干果中加入10 mL水时,各个农药化合物的提取效果最佳。

**图 2:**
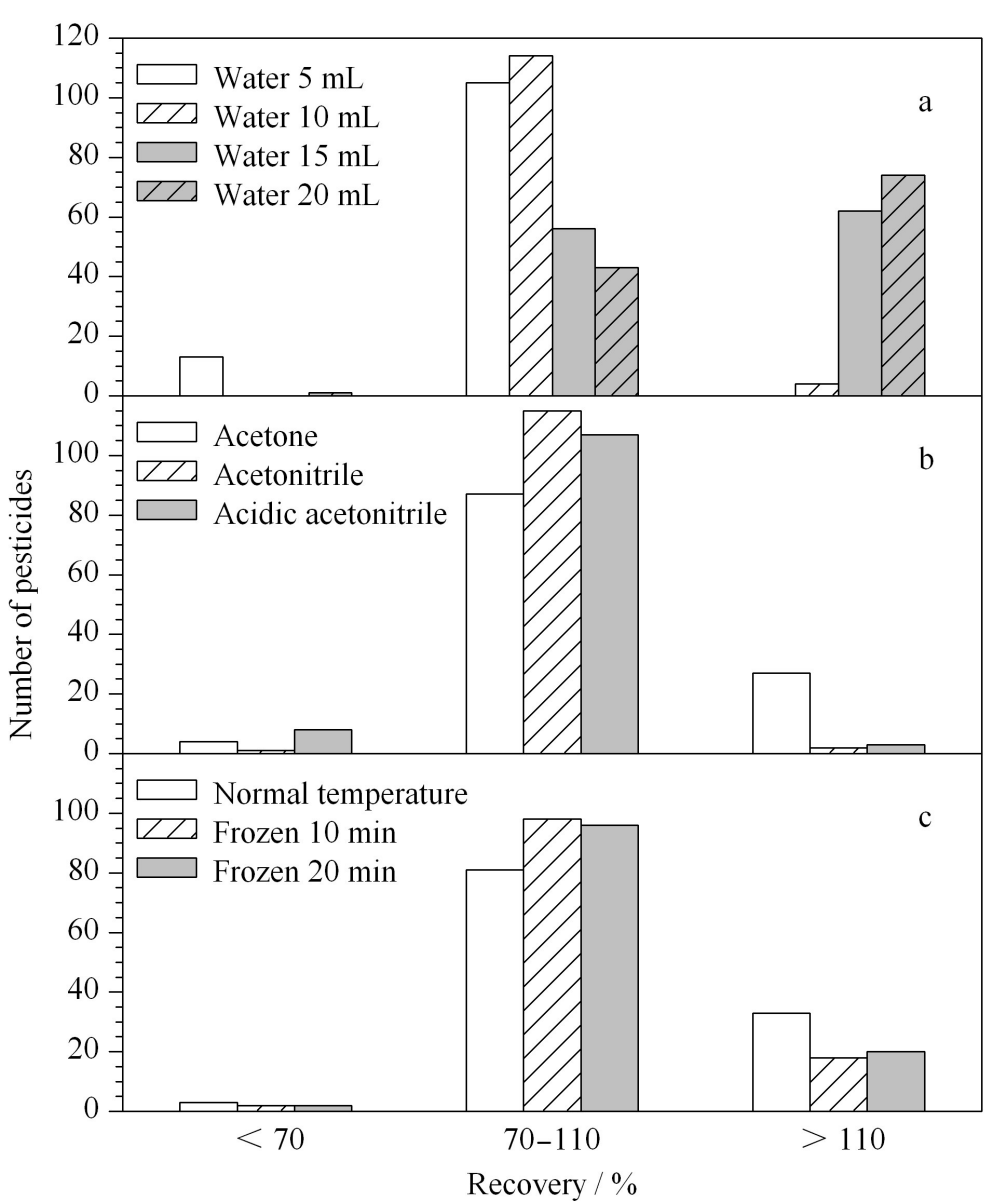
(a)加水量、(b)提取溶剂和(c)提取温度对枸杞干果中118种农药回收率的影响

2.2.2 提取溶剂的筛选

本实验依据国家标准^[16]^中涉及的农药目录以及果蔬中常用农药选择了有机磷、有机氯、三唑类、拟除虫菊酯类和酰胺类等118种农药,由于农药种类多、极性差异不同,因此筛选适合大多数农药的提取溶剂尤为重要。对比了丙酮、正己烷、乙腈和含0.1%甲酸的乙腈(简称为酸化乙腈)4种溶剂提取后的溶液颜色,结果表明丙酮提取液颜色最深,正己烷次之,乙腈和酸化乙腈的提取液颜色相当且较浅(见图3),考虑对质谱仪器的污染,以乙腈和酸化乙腈为宜。同时对目标农药进行了回收率试验,结果显示采用正己烷时,农药的回收率整体水平偏低,可能是因为正己烷极性相对较弱,不利于极性较大农药的提取;其他3种溶剂(见图2b)的农药回收率占比数量结果表明,当提取溶剂依次为乙腈、酸化乙腈、丙酮时,回收率为70%~110%的农药数量逐渐减少;丙酮为提取溶剂时,回收率偏高或偏低的较多;酸化乙腈提取时有部分农药的回收率偏低,可能与农药的结构或酸碱性有关,影响其提取效果。综合考虑,以乙腈为溶剂可满足大多数农药的提取效果。

**图 3:**
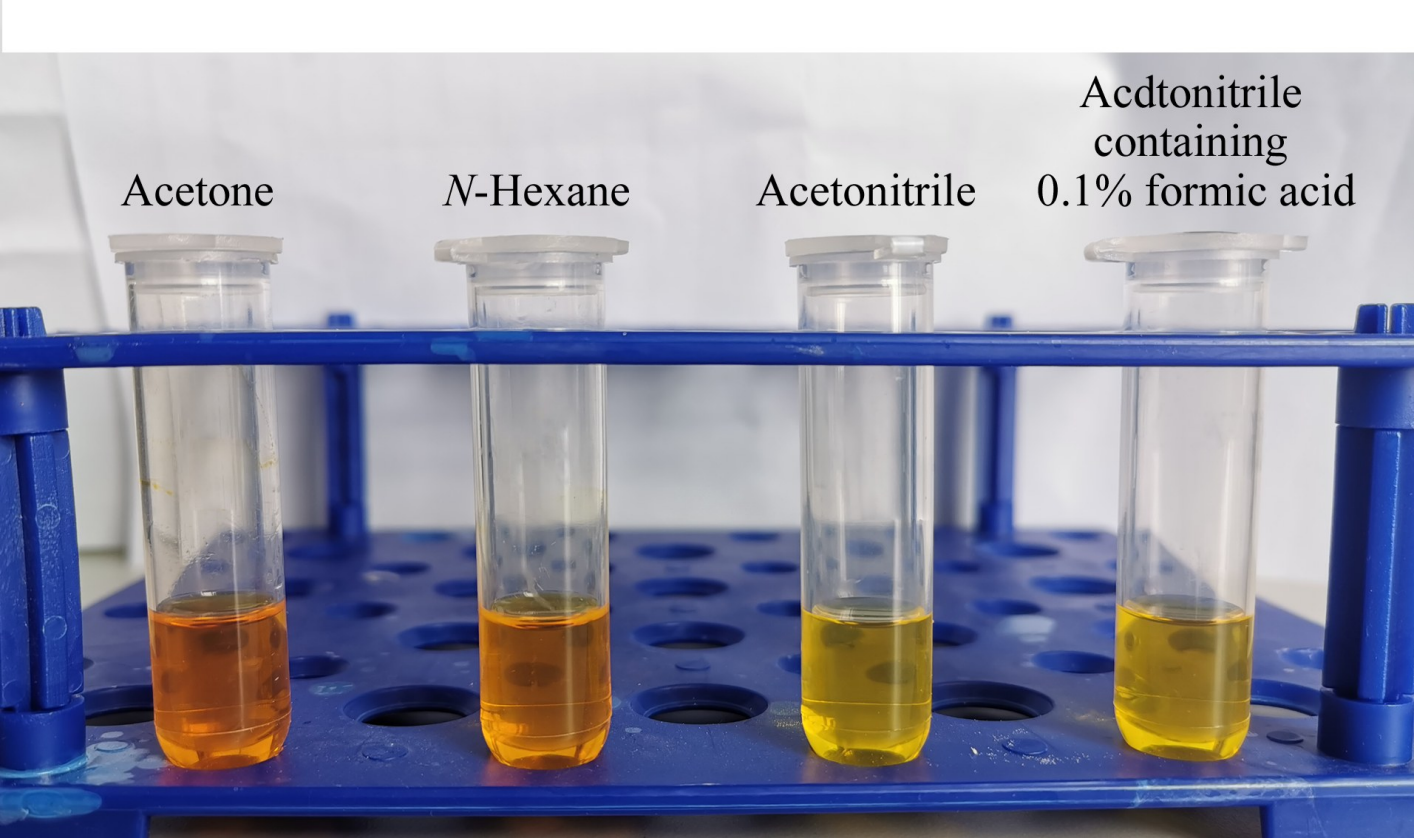
不同溶剂提取液的颜色对比

2.2.3 提取温度的优化

本实验用乙腈溶剂提取后,参照QuEChERS法加入内含4.0 g无水硫酸镁、1.0 g氯化钠、1.0 g柠檬酸钠、0.5 g柠檬酸二钠的提取盐包。氯化钠可促进溶液分层,缓冲体系可有效防止对酸碱敏感农药的降解,无水硫酸镁在提取过程中可吸水,但是吸水会产生大量热量,影响热不稳定农药的回收率。本实验对乙腈提取液分别采取常温、-18 ℃冷冻10 min和20 min后加入缓冲体系提取试剂考察提取过程中温度对提取回收率的影响。从图2c可以看出,常温下回收率大于110%的农药数量最多,-18 ℃冷冻10 min和20 min后农药的回收率在同一范围内的数量相当,从节约时间和经济方面综合考虑,本实验选取-18 ℃冷冻10 min为最佳提取温度。

### 2.3 净化条件的优化

枸杞中主要含多糖、类胡萝卜素等物质,这些物质会随着目标农药被一起提取,净化处理可降低其对目标化合物的干扰。果蔬中常用的净化材料有PSA、C_18_、GCB和吸水剂无水硫酸镁。PSA主要对极性有机酸、糖类和脂类产生吸附;C_18_主要去除脂类、类胡萝卜素和叶黄素等色素;GCB主要去除叶绿素和极性小分子干扰物等色素,但对平面结构目标物具有很强的吸附性^[17,18]^。根据基质中存在的干扰组分和前期实验^[13]^基础,本实验选择无水硫酸镁、PSA、C_18_考察其净化效果。

2.3.1 无水硫酸镁添加量的优化

在PSA 150 mg、C_18_ 100 mg的固定条件下,比较无水硫酸镁用量(400、800、1000、1200 mg)对农药回收率的影响(见图4a)。结果显示,无水硫酸镁用量400 mg时,低回收率农药数量较多;无水硫酸镁用量800 mg时,回收率在70%~120%的农药数量占比最大;无水硫酸镁用量为1000 mg和1200 mg时,偏低和偏高回收率的农药数量居多。综合考虑,选取800 mg为无水硫酸镁添加量。

**图 4:**
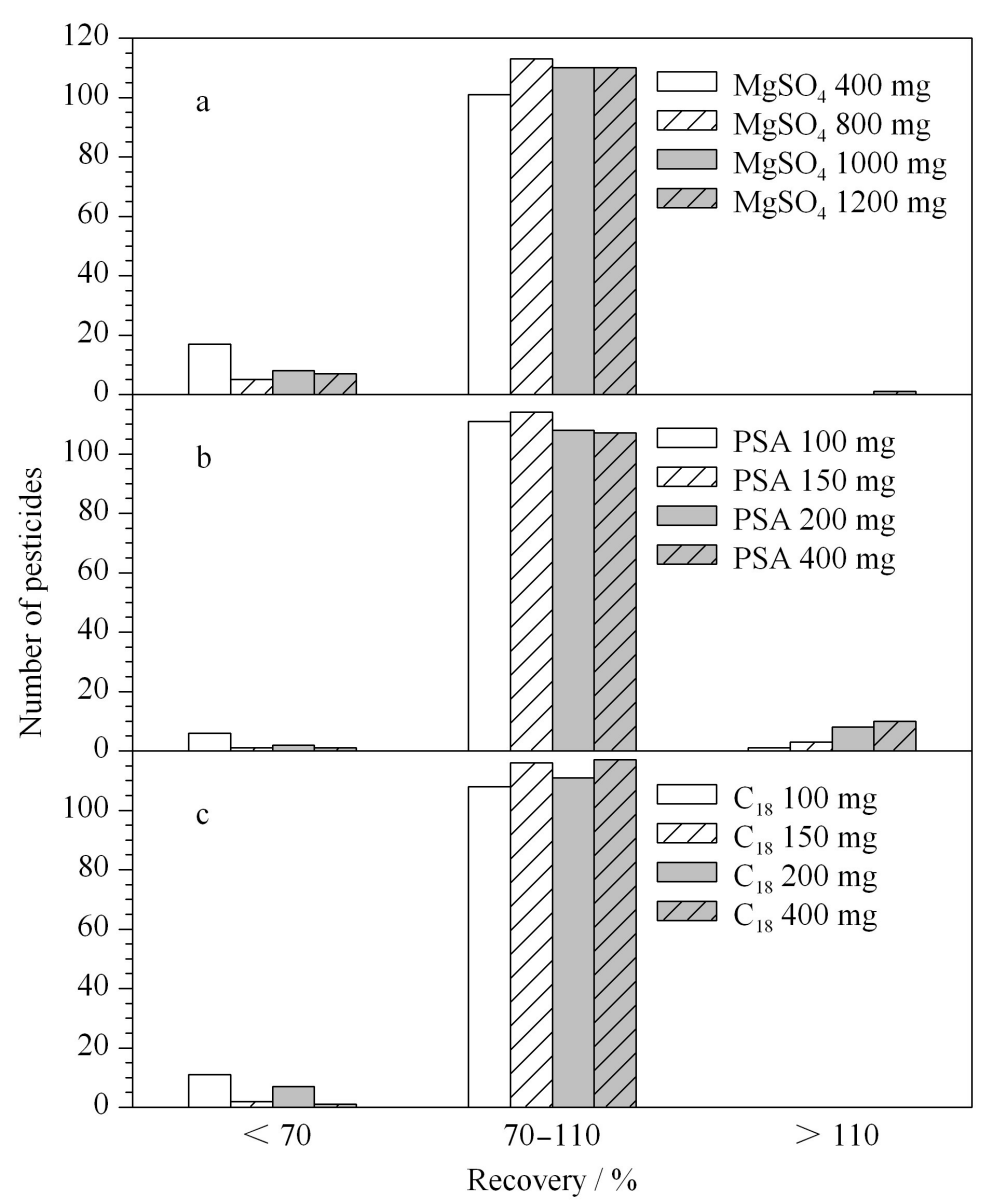
(a)无水硫酸镁、(b)PSA和(c)C_18_用量对枸杞干果中118种农药净化效果的影响

2.3.2 PSA添加量的优化

在无水硫酸镁800 mg、C_18_ 100 mg的固定条件下,对比100、150、200、400 mg PSA对目标农药回收率的影响(见图4b)。从图中可以看出,PSA为100 mg时,低于70%回收率的农药数量最多;当PSA为150 mg时,回收率在70%~120%的农药数量最多,当PSA量超过150 mg时,偏高回收率的农药数量明显增加。故本实验PSA添加量采用150 mg。

2.3.3 C_18_添加量的优化

在无水硫酸镁800 mg、PSA 150 mg的固定条件下,比较100、150、200、400 mg C_18_添加量对目标农药净化效果的影响(见图4c)。结果发现:所有农药回收率均低于120%。C_18_添加量为100 mg和200 mg,回收率小于70%农药数量居多;C_18_为150 mg和400 mg时,同一回收率范围的农药数量占比相当。因此,考虑耗材用量,最终选取150 mg为C_18_添加量。

### 2.4 方法学考察

2.4.1 线性关系、相关系数及检出限

按照1.3.2节方法处理空白枸杞样品,得到空白基质溶液,然后配制成质量浓度为20~640 μg/L的系列基质混合标准溶液,以各个农药的峰面积为纵坐标,质量浓度为横坐标,考察118种农药的线性关系。结果显示,118种农药在相应的浓度范围内具有良好的线性关系,相关系数(*R*^2^)≥0.9923(见表2)。采用空白样品添加方式考察118种农药的检出限(*S/N*=3)和定量限(*S/N*=10),检出限为0.006~28.344 μg/kg,定量限为0.021~94.480 μg/kg,符合多数农药残留筛查要求。

**表 2:** 118种农药的线性方程、相关系数、检出限、定量限、加标回收率和相对标准偏差

No.	Linear equation	R^2^	LOD/ (μg/kg)	LOQ/ (μg/kg)	Recoveries/% (RSD/%) (n=6)								
					0.01 mg/kg	0.04 mg/kg	0.10 mg/kg	0.20 mg/kg
1	y=1.783×10^3^x+4.020×10^3^	0.9992	1.039	3.462	102.90 (3.74)	95.44 (6.32)	92.21 (1.58)	92.42 (1.24)
2	y=1.056×10^3^x+2.858×10^4^	0.9923	0.618	2.061	125.37 (2.60)	117.69 (5.31)	119.85 (1.12)	118.00 (5.26)
3	y=3.849×10^2^x+4.780×10^3^	0.9976	4.012	13.373	111.07 (5.25)	113.49 (6.19)	118.91 (1.75)	118.56 (2.11)
4	y=2.498×10^3^x+6.403×10^4^	0.9944	2.560	8.533	73.82 (7.15)	114.56 (4.20)	99.35 (3.14)	88.20 (10.54)
5	y=4.311×10^3^x+1.842×10^4^	0.9993	7.822	26.073	106.76 (5.83)	103.89 (5.06)	105.63 (1.30)	107.21 (1.62)
6	y=7.049×10^2^x-5.893×10^2^	0.9995	0.070	0.232	103.07 (6.18)	102.63 (2.61)	100.83 (1.91)	107.68 (2.64)
7	y=2.427×10^2^x-1.317×10^2^	0.9986	0.573	1.909	92.99 (9.75)	98.29 (11.53)	100.69 (10.25)	108.69 (10.39)
8	y=6.631×10^3^x+6.141×10^4^	0.9983	0.737	2.458	114.78 (3.34)	105.71 (4.30)	102.36 (1.04)	106.57 (1.35)
9	y=2.317×10^3^x-4.378×10^3^	0.9992	0.309	1.028	96.83 (2.76)	110.59 (4.63)	100.37 (2.70)	108.24 (1.87)
10	y=1.819×10^3^x+1.210×10^4^	0.9979	0.758	2.528	114.56 (2.27)	106.93 (3.16)	103.81 (0.80)	107.6 (0.81)
11	y=2.518×10^3^x-4.333×10^4^	0.9988	0.019	0.064	117.49 (3.21)	101.27 (3.57)	106.98 (1.55)	118.92 (1.91)
12	y=1.581×10^3^x+4.248×10^3^	0.9993	0.481	1.603	112.53 (3.31)	108.47 (2.76)	104.47 (1.57)	108.44 (0.80)
13	y=4.489×10^3^x+1.364×10^5^	0.9993	0.059	0.195	116.38 (2.49)	109.64 (3.34)	105.47 (0.93)	110.93 (1.08)
14	y=9.454×10^2^x+2.810×10^3^	0.9993	0.092	0.306	112.83 (4.32)	105.71 (5.73)	103.51 (0.69)	108.36 (1.52)
15	y=5.135×10^3^x+1.662×10^4^	0.9992	0.056	0.185	110.32 (1.14)	102.91 (3.05)	98.59 (1.98)	102.94 (0.81)
16	y=2.402×10^3^x+1.299×10^4^	0.9987	0.171	0.569	93.93 (4.00)	91.42 (3.00)	88.70 (1.99)	90.71 (1.90)
17	y=4.590×10^2^x+2.942×10^2^	0.9980	28.344	94.480	105.73 (8.65)	97.47 (9.75)	96.34 (2.04)	96.18 (4.56)
18	y=1.830×10^3^x+6.118×10^3^	0.9994	0.273	0.911	109.56 (1.89)	100.90 (3.44)	99.32 (2.38)	103.00 (1.26)
19	y=7.235×10^3^x+1.192×10^4^	0.9993	0.065	0.216	109.84 (1.25)	99.85 (3.61)	96.57 (1.68)	99.75 (0.95)
20	y=3.427×10^3^x+7.406×10^3^	0.9991	0.450	1.499	114.47 (2.88)	107.71 (2.37)	104.25 (1.04)	109.32 (1.43)
21	y=4.960×10^3^x+1.178×10^4^	0.9992	0.599	1.996	112.41 (3.55)	102.96 (6.02)	103.49 (1.49)	104.80 (2.92)
22	y=1.521×10^3^x+1.169×10^4^	0.9987	4.350	14.500	112.82 (2.80)	89.51 (1.80)	96.68 (1.79)	102.90 (1.28)
23	y=8.221×10^2^x-3.979×10^3^	0.9997	0.019	0.063	110.93 (7.81)	114.81 (5.51)	104.11 (1.52)	108.32 (2.02)
24	y=4.268×10^3^x+1.818×10^4^	0.9990	0.775	2.584	105.84 (6.73)	101.14 (6.69)	99.98 (1.91)	107.23 (0.81)
25	y=2.145×10^3^x+1.751×10^4^	0.9970	0.258	0.861	125.11 (2.26)	109.21 (3.19)	102.77 (2.67)	107.15 (1.74)
26	y=1.100×10^3^x+4.281×10^3^	0.9993	0.539	1.797	106.67 (4.27)	104.61 (3.78)	99.22 (1.85)	105.60 (1.32)
27	y=8.925×10^2^x-7.708×10^3^	0.9991	1.102	3.675	95.57 (4.87)	79.61 (2.11)	76.31 (3.64)	83.21 (1.48)
28	y=2.980×10^3^x+1.057×10^4^	0.9991	0.136	0.453	106.44 (2.35)	96.96 (7.85)	99.38 (1.22)	98.16 (4.64)
29	y=1.575×10^3^x+3.597×10^3^	0.9982	1.232	4.107	94.00 (2.50)	71.50 (2.96)	85.03 (1.43)	76.43 (5.05)
30	y=7.810×10^2^x+3.940×10^4^	0.9982	2.295	7.649	119.48 (4.69)	106.51 (2.68)	104.19 (3.03)	107.49 (1.16)
31	y=1.716×10^3^x+4.912×10^3^	0.9989	0.016	0.052	109.51 (4.35)	105.71 (3.72)	100.76 (1.57)	104.49 (1.09)
32	y=9.707×10^2^x-1.496×10^4^	0.9989	3.435	11.451	113.84 (2.43)	106.11 (7.05)	107.48 (1.61)	114.13 (2.32)
33	y=4.320×10^3^x+1.466×10^4^	0.9991	0.167	0.557	117.12 (2.10)	105.83 (2.84)	102.82 (1.74)	105.87 (0.96)
34	y=4.653×10^2^x+2.150×10^4^	0.9993	4.972	16.574	101.01 (5.03)	110.27 (15.43)	105.62 (1.26)	93.00 (6.25)
35	y=2.416×10^3^x-4.850×10^3^	0.9997	0.078	0.259	112.33 (4.72)	100.95 (1.38)	94.97 (2.48)	95.30 (1.17)
36	y=4.494×10^2^x+2.229×10^4^	0.9980	2.017	6.723	89.40 (5.00)	104.23 (13.41)	100.52 (3.50)	101.32 (2.92)
37	y=2.178×10^3^x+8.902×10^3^	0.9993	1.544	5.146	118.79 (2.88)	114.96 (3.71)	103.89 (1.39)	110.86 (0.72)
38	y=1.416×10^3^x-1.355×10^4^	0.9990	0.120	0.401	109.51 (3.35)	106.43 (5.83)	106.54 (1.95)	114.78 (1.54)
39	y=3.369×10^3^x-2.026×10^3^	0.9992	0.133	0.444	113.87 (3.01)	102.83 (4.36)	101.08 (1.04)	104.99 (0.96)
40	y=2.921×10^3^x+1.249×10^4^	0.9986	0.417	1.390	104.63 (3.85)	103.31 (4.59)	103.63 (1.54)	106.01 (1.07)
41	y=8.650×10^3^x+1.556×10^3^	0.9992	0.438	1.460	120.64 (8.50)	105.27 (5.80)	94.11 (3.65)	97.98 (3.40)
42	y=1.562×10^3^x+6.347×10^3^	0.9992	0.011	0.037	119.17 (2.67)	111.57 (2.35)	104.39 (1.11)	107.55 (1.47)
43	y=1.397×10^3^x-2.752×10^4^	0.9984	1.727	5.756	117.83 (2.59)	111.70 (4.89)	114.12 (1.31)	123.24 (1.47)
44	y=1.616×10^3^x+3.949×10^3^	0.9994	5.179	17.262	116.61 (2.13)	100.02 (3.82)	102.41 (1.89)	102.62 (3.75)
45	y=4.595×10^3^x+5.638×10^4^	0.9924	0.345	1.149	104.14 (2.11)	96.52 (5.27)	95.60 (1.65)	93.89 (3.41)
46	y=3.862×10^3^x+1.101×10^4^	0.9984	1.230	4.101	114.06 (3.02)	106.74 (1.91)	100.49 (1.80)	106.12 (3.03)
47	y=5.960×10^3^x+2.804×10^4^	0.9984	0.371	1.238	118.79 (2.02)	106.99 (2.92)	103.80 (1.63)	107.23 (0.93)
48	y=5.924×10^3^x+1.239×10^4^	0.9992	0.196	0.655	108.53 (1.65)	102.95 (3.05)	99.28 (1.85)	105.26 (1.31)
49	y=8.395×10^2^x-1.333×10^4^	0.9978	0.031	0.103	117.07 (3.44)	80.64 (5.58)	103.58 (1.82)	118.20 (2.04)
50	y=3.849×10^3^x+2.270×10^4^	0.9993	0.092	0.307	108.77 (3.89)	100.29 (4.79)	100.91 (1.30)	102.41 (2.62)
51	y=2.194×10^3^x+6.487×10^4^	0.9962	2.256	7.520	106.17 (4.10)	91.39 (4.32)	91.56 (2.92)	93.93 (1.71)
52	y=1.567×10^2^x+4.088×10^4^	0.9942	1.188	3.961	91.11 (9.45)	83.41 (11.59)	87.20 (6.62)	80.22 (4.66)
53	y=2.292×10^3^x+6.655×10^3^	0.9991	1.201	4.004	96.60 (3.64)	89.20 (1.20)	91.59 (1.40)	90.99 (1.36)
54	y=1.023×10^3^x+1.791×10^4^	0.9946	3.993	13.311	126.21 (6.82)	115.09 (4.1)	118.40 (1.61)	119.91 (3.70)
55	y=1.838×10^3^x+4.663×10^3^	0.9995	7.033	23.444	113.47 (3.21)	105.99 (5.22)	102.84 (1.62)	105.33 (2.28)
56	y=1.243×10^3^x+1.081×10^3^	0.9992	2.023	6.742	116.37 (2.96)	101.81 (2.77)	98.61 (2.12)	102.11 (0.74)
57	y=1.965×10^3^x+6.867×10^3^	0.9991	0.699	2.330	112.31 (2.99)	106.55 (4.81)	105.68 (1.34)	108.28 (0.70)
58	y=5.571×10^3^x+1.171×10^4^	0.9984	0.755	2.517	113.35 (3.70)	101.33 (6.77)	100.05 (2.22)	99.28 (2.08)
59	y=7.872×10^1^x+3.365×10^2^	0.9986	1.562	5.208	86.61 (18.86)	111.13 (17.20)	103.43 (6.65)	107.93 (6.24)
60	y=3.459×10^3^x+1.928×10^4^	0.9983	1.291	4.302	103.22 (7.24)	108.98 (4.66)	103.10 (1.76)	105.93 (2.07)
61	y=5.909×10^3^x+2.370×10^4^	0.9991	0.059	0.198	111.09 (2.61)	104.83 (2.57)	99.44 (1.66)	104.20 (1.94)
62	y=2.022×10^3^x-5.380×10^4^	0.9966	0.756	2.520	118.31 (5.46)	113.84 (3.44)	114.45 (1.10)	125.67 (3.03)
63	y=3.948×10^3^x+1.053×10^4^	0.9993	0.278	0.927	117.57 (2.78)	105.58 (2.30)	104.47 (2.22)	104.25 (2.11)
64	y=1.098×10^3^x-7.259×10^3^	0.9993	1.840	6.134	96.21 (4.63)	100.03 (4.15)	101.83 (1.37)	101.85 (1.44)
65	y=4.713×10^2^x+1.101×10^3^	0.9984	3.071	10.238	108.01 (7.61)	100.48 (4.04)	98.03 (1.89)	99.96 (2.57)
66	y=8.782×10^2^x+4.111×10^2^	0.9996	0.706	2.352	114.74 (7.52)	104.90 (3.02)	99.94 (2.08)	101.38 (1.80)
67	y=2.816×10^3^x+7.608×10^3^	0.9991	0.454	1.512	103.76 (17.61)	112.00 (4.13)	103.30 (3.66)	104.17 (2.16)
68	y=4.553×10^3^x+2.014×10^4^	0.9991	0.244	0.814	113.87 (2.44)	103.11 (1.47)	99.04 (2.21)	103.52 (1.54)
69	y=3.994×10^2^x+1.736×10^4^	0.9992	2.981	9.938	102.63 (7.12)	102.15 (4.10)	100.27 (4.29)	103.93 (1.24)
70	y=3.726×10^3^x+5.045×10^4^	0.9994	0.120	0.399	104.06 (3.31)	84.51 (2.22)	93.30 (1.25)	91.82 (1.57)
71	y=1.518×10^3^x-1.437×10^2^	0.9995	0.263	0.877	114.58 (1.28)	107.51 (3.36)	107.78 (1.33)	113.62 (1.98)
72	y=8.973×10^3^x+5.973×10^4^	0.9985	0.169	0.563	108.06 (3.58)	95.67 (2.31)	100.08 (1.44)	100.51 (1.95)
73	y=1.667×10^3^x-1.390×10^4^	0.9990	0.155	0.517	89.20 (6.61)	84.31 (4.86)	95.38 (4.00)	91.27 (3.76)
74	y=5.554×10^3^x-2.622×10^4^	0.9994	0.426	1.421	115.65 (0.95)	88.87 (1.59)	100.37 (1.22)	105.07 (1.02)
75	y=1.605×10^2^x+6.936×10^2^	0.9994	1.327	4.422	116.44 (14.47)	112.15 (1.34)	106.02 (2.47)	106.11 (3.77)
76	y=8.279×10^2^x+5.955×10^2^	0.9991	1.404	4.678	114.14 (6.48)	102.70 (6.54)	99.80 (3.53)	104.80 (1.88)
77	y=9.921×10^3^x+5.135×10^4^	0.9986	0.110	0.368	114.09 (1.49)	111.38 (3.08)	111.48 (1.09)	119.70 (2.45)
78	y=5.270×10^3^x-3.597×10^4^	0.9996	0.428	1.425	113.85 (1.50)	111.94 (3.01)	111.18 (1.15)	119.47 (2.45)
79	y=4.438×10^2^x+3.510×10^2^	0.9962	2.950	9.834	64.97 (3.17)	96.49 (14.41)	107.88 (4.83)	103.33 (5.58)
80	y=1.292×10^3^x-2.156×10^3^	0.9992	2.171	7.238	116.54 (15.42)	103.82 (3.17)	102.02 (2.80)	101.31 (2.29)
81	y=4.055×10^3^x+1.312×10^4^	0.9992	0.925	3.082	112.49 (2.87)	101.99 (3.78)	102.90 (2.24)	104.01 (2.68)
82	y=4.761×10^3^x-2.754×10^3^	0.9990	0.420	1.400	96.30 (1.70)	94.64 (1.86)	84.48 (3.73)	81.17 (2.34)
83	y=3.756×10^3^x+7.650×10^4^	0.9994	0.714	2.381	117.89 (1.83)	107.23 (1.73)	104.60 (1.85)	107.17 (1.49)
84	y=4.849×10^3^x-6.405×10^4^	0.9986	0.466	1.555	109.23 (1.65)	104.21 (5.57)	104.72 (1.13)	104.05 (3.13)
85	y=2.789×10^3^x+2.162×10^3^	0.9995	7.987	26.624	111.43 (2.61)	92.58 (2.23)	79.09 (4.61)	76.10 (3.71)
86	y=2.222×10^3^x+7.196×10^4^	0.9989	0.006	0.021	104.26 (1.60)	96.88 (1.58)	109.32 (1.06)	101.69 (1.08)
87	y=2.187×10^3^x+8.699×10^4^	0.9957	5.325	17.750	89.79 (3.54)	90.95 (8.07)	118.8 (10.19)	102.47 (8.01)
88	y=5.372×10^3^x+1.027×10^4^	0.9991	0.357	1.191	117.01 (1.87)	108.95 (2.09)	104.18 (1.19)	108.11 (1.74)
89	y=3.241×10^3^x+1.249×10^4^	0.9990	3.288	10.959	106.44 (11.96)	103.35 (5.74)	92.28 (2.27)	97.53 (3.08)
90	y=3.380×10^3^x-2.463×10^3^	0.9980	1.148	3.827	93.65 (1.16)	86.71 (3.40)	89.69 (2.89)	89.37 (2.92)
91	y=3.449×10^3^x-3.735×10^4^	0.9979	1.579	5.264	118.88 (3.68)	107.45 (3.93)	104.26 (2.47)	106.96 (1.18)
92	y=2.406×10^3^x+6.967×10^3^	0.9990	1.053	3.509	117.72 (1.80)	109.77 (1.05)	103.10 (1.75)	106.76 (1.66)
93	y=1.348×10^4^x+5.320×10^4^	0.9989	0.215	0.717	98.22 (2.84)	86.30 (2.48)	88.25 (3.02)	88.37 (2.35)
94	y=1.926×10^2^x+1.039×10^3^	0.9965	6.941	23.136	73.53 (8.01)	79.52 (2.55)	84.74 (4.44)	82.67 (2.45)
95	y=9.110×10^2^x+8.991×10^2^	0.9990	1.069	3.564	120.72 (2.35)	109.13 (0.98)	103.99 (2.52)	108.45 (1.53)
96	y=8.179×10^3^x+1.731×10^4^	0.9993	0.445	1.483	108.15 (2.17)	104.06 (2.69)	101.46 (0.89)	105.03 (1.11)
97	y=3.406×10^3^x-1.262×10^4^	0.9992	2.701	9.004	108.77 (2.29)	98.42 (3.53)	96.80 (2.40)	100.77 (1.42)
98	y=8.037×10^3^x+3.163×10^3^	0.9984	0.391	1.304	116.44 (5.86)	108.71 (2.33)	104.28 (2.02)	108.62 (1.28)
99	y=3.451×10^3^x-4.254×10^4^	0.9987	0.325	1.083	104.86 (1.51)	94.40 (1.01)	90.54 (2.16)	94.73 (1.60)
100	y=5.655×10^3^x+5.295×10^3^	0.9996	0.061	0.204	112.41 (2.33)	88.32 (2.21)	85.56 (2.87)	96.26 (3.27)
101	y=3.775×10^3^x+1.038×10^4^	0.9988	2.055	6.850	115.73 (4.94)	104.8 (2.34)	101.24 (2.61)	105.56 (3.60)
102	y=2.693×10^3^x+1.005×10^4^	0.9986	19.103	63.676	79.37 (8.39)	109.58 (3.80)	103.82 (3.12)	110.12 (2.75)
103	y=1.711×10^4^x-1.296×10^2^	0.9990	0.285	0.948	116.57 (0.70)	107.77 (2.00)	104.66 (2.08)	109.86 (1.64)
104	y=8.777×10^2^x-3.046×10^3^	0.9984	1.523	5.076	108.64 (2.24)	105.24 (4.46)	106.90 (2.55)	105.78 (2.55)
105	y=9.344×10^2^x+1.851×10^3^	0.9992	0.561	1.869	100.47 (1.53)	72.07 (2.74)	94.45 (1.83)	96.95 (2.08)
106	y=4.959×10^3^x-1.057×10^4^	0.9990	0.483	1.611	99.06 (2.81)	70.73 (9.21)	83.91 (1.67)	84.14 (2.52)
107	y=1.958×10^3^x-6.015×10^3^	0.9995	3.955	13.184	108.95 (3.12)	105.44 (2.52)	100.76 (2.05)	107.57 (3.25)
108	y=6.608×10^3^x+6.562×10^3^	0.9989	0.263	0.877	103.31 (1.37)	85.13 (5.39)	97.15 (0.77)	93.49 (5.49)
109	y=2.536×10^3^x-5.407×10^3^	0.9993	4.058	13.527	106.31 (2.72)	111.61 (3.73)	101.33 (6.70)	115.99 (3.47)
110	y=1.441×10^3^x+4.545×10^3^	0.9984	0.439	1.464	113.49 (4.82)	108.85 (4.27)	108.93 (1.07)	110.91 (1.66)
111	y=3.343×10^3^x-1.637×10^4^	0.9992	2.885	9.615	116.30 (2.26)	102.14 (2.26)	103.71 (2.81)	111.04 (2.63)
112	y=1.574×10^4^x+4.662×10^4^	0.9988	0.201	0.670	116.99 (1.09)	108.89 (1.08)	103.05 (2.04)	107.62 (2.04)
113	y=2.760×10^3^x-4.175×10^4^	0.9985	0.891	2.971	105.51 (3.35)	96.44 (2.71)	92.24 (2.63)	101.53 (2.97)
114	y=2.465×10^3^x-1.774×10^4^	0.9993	2.420	8.068	98.37 (4.35)	90.12 (1.90)	89.82 (2.28)	108.32 (5.75)
115	y=5.389×10^3^x+7.418×10^3^	0.9984	3.300	11.000	112.28 (2.67)	98.82 (1.86)	106.20 (1.26)	106.93 (1.58)
116	y=1.145×10^3^x-1.125×10^4^	0.9992	6.258	20.860	98.30 (4.30)	83.58 (5.19)	87.34 (2.32)	103.89 (5.30)
117	y=7.157×10^2^x+2.269×10^3^	0.9974	0.685	2.283	100.43 (5.08)	69.7 (4.06)	90.04 (2.07)	82.55 (4.73)
118	y=3.245×10^3^x+8.898×10^3^	0.9977	0.231	0.769	97.77 (1.99)	68.77 (5.83)	84.65 (1.81)	81.02 (1.57)

Nos. 1-118 are the same as that in Table 1. *y*: peak area; *x*: mass concentration, μg/L.

2.4.2 加标回收率与精密度

以空白枸杞样为基质,添加0.01、0.04、0.10、0.20 mg/kg 4个水平的混合标准溶液,每个加标水平做6次平行实验,采用优化后的条件测定。结果显示,118种农药平均回收率为64.97%~126.21%,RSD为0.69%~18.86%。可见,该方法的准确度和精密度较高,可用于测定样品中多种农药残留。

### 2.5 基质效应的考察

基质效应(ME)是空白基质匹配校正曲线的斜率/溶剂标准曲线的斜率-1。当|ME|小于20%时,表示弱基质效应可忽略不计;|ME|为20%~50%时,表示中等基质效应;|ME|大于50%时表示强基质效应。结果表明,82%农药的ME值为正值,呈现为基质增强作用,18%的农药呈现基质抑制作用(见图5)。74%的农药表现出较弱的基质效应,17%的农药具有中等效应,只有9%的农药为强基质效应。因此本实验采用基质匹配标准曲线,可降低对目标农药基质效应的影响。

**图 5:**
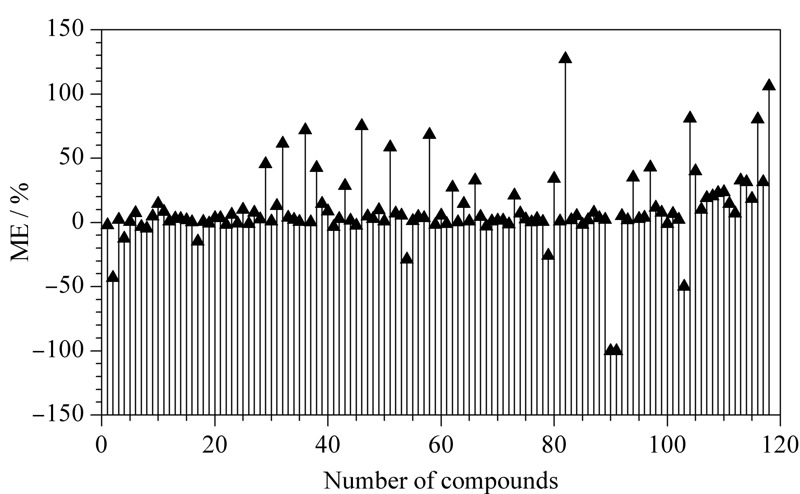
118种农药在枸杞干果中的基质效应

### 2.6 样品的测定

采用建立的方法测定了10批市售枸杞样品中118种农药残留,全部样品均有农药检出,单批样本中检出农药种类3~12种,共检出农药22种,检出率较高的有毒死蜱、氟虫腈、氯氰菊酯、哒螨灵和苯醚甲环唑,其中有一批样本中克菌丹含量达到1.4066 mg/kg(见表3)。可见枸杞在种植过程中农药使用情况普遍存在,且使用的农药种类较多,今后需要相关部门加强监管,严格控制枸杞生产过程中的用药种类和剂量,提高产品质量,保障用药饮食安全。

**表 3:** 10批样品农药残留的测定结果

Compound	Number of detected pesticides	Content/(mg/kg)	
Carbofuran	3	0.0119	-0.1700
Captan	2	0.1390	-1.4066
Chlorpyrifos	9	0.0039	-0.1553
Triadimefon	1	0.0163	
Isocarbofos	2	0.0097	-0.1600
Fipronil	3	0.0121	-0.0420
Triadimenol	2	0.0207	-0.2558
Hexaconazole	1	0.1210	
Myclobutanil	1	0.0357	
Fipronil	6	0.0012	-0.0115
Chlorfenapyr	1	0.0560	
Triazophos	1	0.0085	
Tebuconazole	3	0.0331	-0.3620
Propargite	2	0.0704	-0.1007
Bifenthrin	4	0.0014	-0.0154
Fenpropathrin	4	0.0045	-0.0199
Pyriproxyfen	1	0.0024	
Cypermethrin	5	0.0097	-0.0591
Pyridaben	5	0.0084	-0.0650
Fenvalerate	2	0.0449	-0.0669
Difenoconazole	5	0.0077	-0.1864
Dimethomorph	1	0.0055	

## 3 结论

本文利用气相色谱-三重四极杆质谱动态多反应监测模式,建立了枸杞干果中多种农药残留的高通量检测方法,该方法前处理操作简便、快速,基质匹配标曲法定量准确,通过对实际样品的测定,证实了该方法具有高效、准确、重复性好的优点,适用于枸杞中农药多残留的快速筛查与定量检测,可为市场监管的日常检验和产品质量的风险评估提供技术支撑。
